# Government-administered benefit programs: missed opportunities to address poverty in the NICU

**DOI:** 10.1038/s41372-025-02409-8

**Published:** 2025-09-10

**Authors:** John Feister, Emily R. Miller, Ndidi Unaka, Andrew F. Beck

**Affiliations:** 1 Department of Pediatrics, University of Cincinnati College of Medicine, Cincinnati, OH USA; 2 Division of Neonatology, Perinatal Institute, Cincinnati Children’s Hospital Medical Center, Cincinnati, OH USA; 3 Michael Fisher Child Health Equity Center, Cincinnati Children’s Hospital Medical Center, Cincinnati, OH USA; 4 Department of Pediatrics, Stanford University School of Medicine, Palo Alto, CA USA; 5 Stanford Medicine Children’s Health, Palo Alto, CA USA; 6 Divisions of General & Community Pediatrics and Hospital Medicine, Cincinnati Children’s Hospital Medical Center, Cincinnati, OH USA; 7 Office of Population Health, Cincinnati Children’s Hospital Medical Center, Cincinnati, OH USA

**Keywords:** Health policy, Paediatrics

## Background

Poverty, and associated financial strain, disproportionately affects families with children in the neonatal intensive care unit (NICU), resulting in multigenerational effects on infant/family health and well-being [1]. Numerous effective government-administered benefits programs combat poverty and its effects but are underutilized by eligible families [2], representing thousands of dollars per family per year in unclaimed benefits. We sought to determine eligibility for and successful receipt of government benefit programs for families with infants hospitalized at a large and well-supported level IV NICU in Ohio.

## Methods

We approached families in a one-month period with children in the NICU identified by social workers as potentially eligible for any government benefit program. Roughly 60% of families in this NICU use Medicaid as their primary payor for hospitalization. Most patients are transferred from an outside hospital.

We asked families to complete an electronic survey (Supplemental Instrument 1) focusing on utilization and barriers to utilization of government benefits. Participation was compensated with a $50 gift card. Benefits of interest included refundable tax credits (Earned Income Tax Credit (EITC)), nutrition assistance (Supplemental Nutritional Assistance Program (SNAP) and the Special Supplemental Nutrition Program for Women, Infants, and Children (WIC)), home utility assistance (Low Income Home Energy Assistance Program (LIHEAP)), and childcare payment vouchers. We collected demographic information, including income, to estimate eligibility for each program using state-specific criteria. We also queried interest in receiving in-hospital-based assistance to help families access these benefits. The survey explained each benefit, and families received a United Way resource to help identify and access eligible benefits. We used descriptive statistics to enumerate survey responses.

## Results

25 out of 33 families approached completed surveys. Sociodemographic characteristics are described in Supplemental Table 1. The sample median annual household income was estimated to be $49 500 (190% of the federal poverty line for the sample median household size of 3); 70% of all families surveyed were estimated to be eligible for at least one benefit. Most families who were estimated eligible reported not receiving benefits (Table 1; 27% EITC, 73% WIC, 12% SNAP, 82% LIHEAP, 33% childcare vouchers). Families commonly cited lack of awareness and difficulty completing applications as reasons why they had not applied for benefits (Table 1). Among those who filed taxes in the previous year, 12/23 (52%) reported using a paid tax preparation service (median fee = $200). Finally, 22/25 (88%) of families reported that they would likely use a hospital-based program designed to help with claiming government benefits.

**Table 1:** Percent of families likely eligible for each benefit who reported receiving said benefit.

	EITC	WIC	SNAP	LIHEAP	Childcare Voucher
Among those surveyed: likely eligible (%)	***n*** = 25	***n*** = 25	***n*** = 25	***n*** = 25	***n*** = 25
likely	15 (60)	11 (44)	8 (32)	11 (44)	3 (12)
unlikely	8 (32)	12 (48)	15 (60)	12 (48)	3 (12)
not needed^a^	NA	NA	NA	NA	19 (76)
uncertain^b^	2 (8)	2 (8)	2 (8)	2 (8)	NA
Among those likely eligible: received (%)	***n*** = 15	***n*** = 11	***n*** = 8	***n*** = 11	***n*** = 3
yes	8 (53)	3 (27)	7 (88)	2 (18)	2 (67)
no	4 (27)	8 (73)	1 (12)	9 (82)	1 (33)
not sure	2 (13)	NA	NA	NA	NA
missing response	1 (6)	NA	NA	NA	NA
Among those likely eligible and who did not receive: applied (%)	***n*** = 4	***n*** = 8	***n*** = 1	***n*** = 9	***n*** = 1
yes	4 (100)	3 (38)	0 (0)	2 (22)	0 (0)
no	0 (0)	5 (63)	1 (100)	7 (78)	1 (100)
Among those likely eligible who did not receive and who did not apply: reasons for not applying^**c**^ **(%)**	***n*** = **0**	***n*** = **5**	***n*** = **1**	***n*** = **7**	***n*** = **1**
too difficult to apply	NA	4 (80)	NA	NA	NA
did not think would qualify	NA	1 (20)	1 (100)	3 (43)	NA
unaware	NA	NA	NA	4 (57)	NA
do not need or do not want assistance	NA	NA	NA	1 (14)	1 (100)

Eligibility was determined for the period prior to the birth of the hospitalized infant.

*EITC* Earned Income Tax Credit, *WIC* Special Supplemental Nutrition Program for Women, Infants, and Children, *SNAP* Supplemental Nutritional Assistance Program, *Energy Assistance* Low Income Home Energy Assistance Program.

^a^i.e., do not use childcare.

^b^2 families did not report income, limiting determination of eligibility.

^c^Multiple choices allowed, hence not all columns add to 100%.

## Discussion

Families with infants in the NICU are frequently eligible for government benefit programs but do not uniformly receive them. These benefits represent thousands of dollars in unrealized support for families. Barriers to receiving benefits include lack of awareness and difficulty completing applications. These barriers may grow given current government policy reform. Families express interest in additional help with accessing benefits.

NICUs are positioned to assist families with claiming of government benefits. More broadly, medical financial partnerships are ideally suited for the NICU given their life course effects. As an example, we recently piloted an intervention in collaboration with the United Way of Greater Cincinnati to provide free tax preparation assistance [3] to families with children in the hospital (rather than a primary care setting [4, 5]), proving feasibility. Refundable tax credits are a form of cash transfer, an anti-poverty intervention with a growing body of literature [6]. We prepared 21 tax returns (majority of clients were without hospitalized children), assisting families by saving roughly $300 in preparation fees and claiming around $900 in refunds per return. Future work will expand on using this collaboration as a model to assist families in accessing other government-administered benefit programs.

An important limitation to this work is that our sample was drawn in a one-month period from a population at a NICU that serves as a regional and national referral center. We may under or overestimate the eligibility for and claiming of benefits by families in other NICUs of differing sociodemographic characteristics. Furthermore, although our instrument was offered in multiple languages, we were unable to recruit any families with preferred language other than English. Additionally, as no validated survey instrument exists for this purpose, our instrument was created de novo. Our instrument was reviewed and approved for content and readability by both community members and content experts.

In conclusion, many families in the NICU do not claim anti-poverty government benefit programs. Interventions to enhance successful receipt of government benefit programs offer promise to combat poverty for families with hospitalized children and improve infant/family well-being.

## Supplementary information

Supplemental Instrument 1

Supplemental Table 1
