# Supplementary material for: Government-administered benefit programs: missed opportunities to address poverty in the NICU
Source: J Perinatol. 2025 Sep 10;46(10):1959–60. doi: 10.1038/s41372-025-02409-8 (PMC13680269; doi:10.1038/s41372-025-02409-8)
Supplement: Supplementary file 1 [file 41372_2025_2409_MOESM1_ESM.pdf]

Government Benefits for Families in the NICU

Please complete the survey below.

Thank you!

The following survey asks about any government provided benefits you may be using or eligible for. The results of this survey will help us better support families in the NICU. Thank you in advance for your time!

Have you heard of the Child Tax Credit?

The Child Tax Credit program can provide a monetary refund for each child you claim on your taxes.

- ◯ Yes
- ◯ No

Have you heard of the Earned Income Tax Credit?

The Earned Income Tax Credit is a government program that gives money to people who work based on how much money they make and how many kids they have.

- ◯ Yes
- ◯ No

Do you know if you received the Earned Income Tax Credit last year?

- ◯ Yes
- ◯ No
- ◯ Not sure

Did you (or someone on your behalf) file taxes last year?

- ◯ Yes
- ◯ No
- ◯ Not sure

If you did not file taxes last year, why not?

**Again, this is confidential. We won't share your answer with anyone else.

- ☐ I did not have enough income to file taxes
- ☐ Not enough time
- ☐ Concern about immigration status
- ☐ Didn't know how
- ☐ Process was too complicated
- ☐ Other (please describe):

____

Who prepared your taxes last year?

- ◯ Self or family member
- ◯ Paid tax preparation (H&R block, private accountant/lawyer, etc)
- ◯ Free tax preparation site (VITA) (e.g. at a community center, school, or other local gathering place)
- ◯ Not sure

If you paid to have your taxes done, approximately how much did you pay?

____

Have you ever used a free tax preparation service?

- ◯ Yes
- ◯ No
- ◯ Not sure

Thinking about years where you decided not to use a free tax preparation service but still filed taxes, please tell us why (mark any that apply):	☐ n/a - I always use free tax preparation services<br>☐ I didn't know free tax preparation existed<br>☐ I couldn't get transportation to a free tax preparation clinic<br>☐ I couldn't go to a free tax preparation clinic when it was open<br>☐ I don't trust free tax preparation services<br>☐ I prefer to use a paid service for tax preparation<br>☐ I filed my taxes on my own without paid help<br>☐ Other (please describe):
	__________________________________
Would you be interested in using a free tax preparation service if it was offered by the hospital during your child's hospitalization?	◯ Yes<br>◯ No
Have you heard of supplemental security income (SSI)?<br><br>**Supplemental Security Income (SSI) is a program from the Social Security Administration that can provide monthly income support and Medicaid coverage for babies in the Neonatal Intensive Care Unit (NICU)**	◯ Yes<br>◯ No
Have you or anyone in your household applied for SSI since your infant was born?	◯ Yes<br>◯ No
If you or someone in your household did apply for SSI, did you receive SSI benefits?	◯ Yes<br>◯ No<br>◯ My application is still being processed
If you have not applied for SSI, why? (select all that apply)	☐ I have never heard of SSI<br>☐ I did not think my family would qualify for SSI<br>☐ We don't need SSI benefits<br>☐ The SSI application process is too difficult<br>☐ Other (please describe):
	__________________________________
Have you heard of the Special Supplemental Nutrition for Women, Infants and Children (WIC)?<br><br>**WIC is a program that helps pregnant women, new moms, and kids under five with low income get healthy food, learn about nutrition, get support for breastfeeding, and find healthcare services.**	◯ Yes<br>◯ No
Do you or anyone in your household receive benefits from WIC?	◯ Yes<br>◯ No

Have you or anyone in your household applied for WIC in the last year?	◯ Yes<br>◯ No
If you or someone in your household did apply for WIC, did you receive WIC benefits?	◯ Yes<br>◯ No
If you have not applied for WIC, why? (select all that apply)	☐ I have never heard of WIC<br>☐ We don't need WIC<br>☐ The WIC application process is too difficult or the wait times are too long.<br>☐ I can't get to the WIC clinic (no transportation, time, or it interferes with my schedule)<br>☐ Stores that accept WIC are too far away or WIC foods are hard to find.<br>☐ Using WIC at the store is embarrassing, uncomfortable, or takes too long.<br>☐ We already get food from another source or don't like the foods provided by WIC.<br>☐ Other (please describe):
	______________________________
Have you heard of the Supplemental Nutrition Assistance Program (SNAP, previously known as the food assistance program or food stamps)?<br><br>**SNAP is a program that helps people with low income buy food by giving them money each month on a special card to use at grocery stores.**	◯ Yes<br>◯ No
Do you or anyone in your household receive benefits from SNAP (in the last year)?	◯ Yes<br>◯ No
Have you or anyone in your household applied for SNAP in the last year?	◯ Yes<br>◯ No
If you or someone in your household did apply for SNAP, did you receive SNAP benefits?	◯ Yes<br>◯ No<br>◯ The application is still being processed

If you have not applied for SNAP, why? (select all that apply)	☐ I did not think I or the person in my household would qualify for SNAP<br>☐ I or the person in my household don't need SNAP<br>☐ I have never heard of SNAP<br>☐ The SNAP application was difficult<br>☐ SNAP foods are difficult to find in the grocery store<br>☐ Using SNAP at the grocery store is embarrassing/uncomfortable<br>☐ Checking out at the store with SNAP foods can take a long time<br>☐ My family already receives food from WIC, a foodbank, or other source<br>☐ I or my family would not like the foods provided by SNAP<br>☐ I do not want myself or my family to receive government benefits<br>☐ Other (describe)
	__________________________________
Have you heard of the Low Income Home Energy Assistance Program (LIHEAP) or other programs to help families pay for utility/heating costs?<br><br>**LIHEAP is a program that helps families with low income pay their heating and cooling bills to keep their homes comfortable and safe**	◯ Yes<br>◯ No
Have you or anyone in your household received benefits from LIHEAP or other programs that help families pay utility bills in the last year?	◯ Yes<br>◯ No
Have you or anyone in your household applied for LIHEAP or other utility assistance in the last year?	◯ Yes<br>◯ No
If you or someone in your household did apply for LIHEAP or other utility assistance, did you receive it?	◯ yes<br>◯ no<br>◯ the application is still being processed
If you have not applied for LIHEAP or other utility assistance, why? (choose all that apply)	☐ I did not think my household would qualify<br>☐ My household doesn't need utility assistance<br>☐ I have never heard of LIHEAP or other utility assistance programs<br>☐ The application was difficult<br>☐ I do not want myself or my family to receive government benefits<br>☐ Other (describe)
	__________________________________

Do you have any children in your family who are cared for by someone other than you in a place other than your home (examples: day care, after school programs)?	◯ Yes<br>◯ No
Have you heard of child care assistance programs or vouchers that help pay for child care?	◯ Yes<br>◯ No
Have you or anyone in your household received child care vouchers or other assistance paying for child care?	◯ Yes<br>◯ No
Have you or anyone in your household applied for child care vouchers or other assistance in the last year?	◯ Yes<br>◯ No
If you or someone in your household did apply for child care vouchers or assistance, did you receive it?	◯ Yes<br>◯ No<br>◯ The application is still being processed
If you have not applied for child care vouchers or assistance, why not? (select all that apply)	☐ I did not think my household would qualify<br>☐ My household doesn't need child care assistance<br>☐ I have never heard of child care assistance<br>☐ The application was difficult<br>☐ I do not want myself or my family to receive government benefits<br>☐ Other (describe)
	__________________________________

Our team is designing a clinic at the hospital to help families get the benefits they qualify for. At the clinic, someone will sit with you to figure out what programs you can get help from, like food, financial, or health assistance. They will also help you fill out the forms to apply for these programs.

Do you think that a benefits application clinic would be helpful to you?	◯ Yes<br>◯ No
If a benefits application clinic was currently offered by the hospital, do you think you would use it?	◯ Yes<br>◯ No
What other things to help with claiming benefits would be useful?	__________________________________

The next few questions are to help us determine which benefits you may be eligible for. They will be used for research purposes only and are strictly confidential.

How many months has your child been hospitalized in the NICU?	◯ < 1 ◯ 1 ◯ 2 ◯ 3 ◯ 4 ◯ 5 ◯ 6 ◯ >6
How old are you?	__________________
Are you married?	◯ Yes<br>◯ No
What state do you live in?	◯ Ohio<br>◯ Kentucky<br>◯ Indiana<br>◯ West Virginia<br>◯ Other (specify)
	__________________
How many people lived in your household in 2023? (The children do not have to be biologically yours. They can be adopted, foster, grandchild, or a relative if they live with you and you claim them on your taxes. Do not Include your newborn infant unless they were born in 2023)	__________________<br>(total number includes yourself)
How many of your household members are children (age 0-18)?	__________________
Are you Hispanic or Latino?	◯ Yes<br>◯ No
What is your race? You may give more than one answer.	☐ White<br>☐ Black Or African American<br>☐ American Indian Or Native American<br>☐ Asian<br>☐ Native Hawaiian Or Other Pacific Islander<br>☐ Other Race
What language do you prefer to communicate in?	◯ English<br>◯ Spanish<br>◯ French<br>◯ Uzbek<br>◯ Nepali<br>◯ Other (specify)
	__________________
What is the highest level of education that you have completed?	◯ Less than High School<br>◯ High School (9th - 12th Grade)<br>◯ Some College<br>◯ College<br>◯ Graduate Degree
Were you or someone in your household employed during the year of 2023?	◯ Yes<br>◯ No

Are you or someone in your household enrolled in Medicaid?	◯ Yes<br>◯ No
What was your household's approximate monthly income last year?<br><br>Income means money you or other adults in your household get from a job, from your own business, pensions, unemployment insurance, cash benefits from assistance programs, alimony or child support. Do not include non-cash benefits like SNAP (food stamps).	◯ No income<br>◯ Less than $1,200 per month (Less than $14,400 per year)<br>◯ $1,200 to $1,500 per month ($14,400 to $18,000 per year)<br>◯ $1,500 to $2,000 per month ($18,000 to $24,000 per year)<br>◯ $2,000 to $2,500 per month ($24,000 to $30,000 per year)<br>◯ $2,500 to $3,500 per month ($30,000 to $42,000 per year)<br>◯ $3,500 to $4,000 per month ($42,000 to $48,000 per year)<br>◯ $4,000 to $4,500 per month ($48,000 to $54,000 per year)<br>◯ $4,500 to $5,000 per month ($54,000 to $60,000 per year)<br>◯ $5,000 to $7,500 per month ($60,000 to $90,000 per year)<br>◯ $7,500 to $10,000 per month ($90,000 to $120,000 per year)<br>◯ Greater than $10,000 per month (greater than $120,000 per year)<br>◯ I am not sure what our approximate monthly income is<br>◯ Prefer not to answer this question
Do you have any money saved in a bank account?	◯ yes<br>◯ no<br>◯ prefer not to answer
Approximately how much money do you have saved in the account?	◯ less than $500<br>◯ $500 to $2000<br>◯ $2000 to $3000<br>◯ More than $3000<br>◯ prefer not to answer
