# Supplementary material for: Government-administered benefit programs: missed opportunities to address poverty in the NICU
Source: J Perinatol. 2025 Sep 10;46(10):1959–60. doi: 10.1038/s41372-025-02409-8 (PMC13680269; doi:10.1038/s41372-025-02409-8)
Supplement: Supplementary file 2 [file 41372_2025_2409_MOESM2_ESM.docx]

**Supplemental table 1**

| Race^ [N (%)] |  |
| --- | --- |
| Black | 5 (20.8) |
| Black, Native American | 1 (4.2) |
| Other | 2 (8.3) |
| White | 15 (62.5) |
| White, Black | 1 (4.2) |
| Ethnicity^ [N (%)] |  |
| Hispanic | 3 (12.5) |
| Non-Hispanic | 21 (87.5) |
| Preferred language [N (%)] |  |
| English | 25 (100.0) |
| Highest level of education completed [N (%)] |  |
| High School (9th - 12th Grade) | 7 (28.0) |
| Some College | 6 (24.0) |
| College | 10 (40.0) |
| Graduate Degree | 2 (8.0) |
| State of residence [N (%)] |  |
| Ohio | 17 (68.0) |
| Indiana | 1 (4.0) |
| Kentucky | 6 (24.0) |
| West Virginia | 1 (4.0) |
| Employed prior to hospitalized infant's birth [N (%)] |  |
| Yes | 24 (96.0) |
| No | 1 (4.0) |
| Anyone in household enrolled in Medicaid [N (%)] |  |
| Yes | 17 (68.0) |
| No | 8 (32.0) |
| Married [N (%)] |  |
| Yes | 15 (60.0) |
| No | 10 (40.0) |
| Age of parent [mean (SD)] | 30.3 (4.8) |
| People in household# [mean (SD)] | 3.1 (1.2) |
| Children under 18 in household [mean (SD)] | 1.8 (1.9) |
| Yearly household Income* [median (range)] | 49,500 (<14,400, >120,000) |
| Length of stay ≧3 months [N (%)] | 16 (64.0) |
| Likely to receive any benefit* [N (%)] | 16 (70.0) |

Supplemental table 1. Selected sociodemographic characteristics of the sample. ^: missing 1 response; #: does not include infant hospitalized; *:2 missing responses
